# Beyond cohorts: how age-based differences on work engagement challenge generational determinism among Spanish professionals

**DOI:** 10.3389/fsoc.2026.1715524

**Published:** 2026-03-25

**Authors:** Maria Claudia Scurtu-Tura, Annabel Jiménez-Soto, Eduardo Infante-Rejano

**Affiliations:** Department of Social Psychology, University of Seville, Seville, Spain

**Keywords:** employee well-being, generational cohorts, intergenerational dynamics, organizational strategies, sociodemographic variables, work engagement

## Abstract

**Introduction:**

Underpinned by the Job Demands-Resources (JD-R) model, this study examines the effectiveness of generational classifications in predicting work engagement, challenging the assumption that birth cohorts determine professional behavior.

**Methods:**

Using a quantitative research approach with a descriptive, cross-sectional design, the study targeted the broad population of active professionals across the Spanish labor market. A final sample of 339 participants (comprising Baby Boomers, Generation X, Millennials, and Generation Z) was selected via non-probabilistic snowball sampling. Primary data were collected using an online questionnaire incorporating the Utrecht Work Engagement Scale (UWES-17).

**Results:**

The results revealed significant differences in the vigor dimension with Baby Boomers scoring higher. Yet they also showed that sociodemographic variables (such as employment status) were more predictive than generational membership.

**Discussion:**

In theoretical terms, this study contributes to the field by demonstrating that the resource-accumulation perspective of the JD-R model is a more effective explanatory framework than “generational determinism.” Empirically, the study provides evidence from the Spanish context that organizational HR strategies should prioritize individual life stages and resource management over broad generational stereotypes.

## 1 Introduction

In today's business environment, companies are required to manage multiple generations of employees, each with their own unique attitudes, values, and work styles. Consequently, one of the most challenging issues for leaders is how to supervise members with different socialization processes who must achieve high level of performance in coordinated efforts (Gurbuz and Aytekin, 2020). For many years, researchers have been fascinated by generational differences in the workplace, yielding many studies that compare different generational cohorts on various work-related variables, such as leadership, teamwork, personality, work attitudes, work-life balance, and career patterns (Rudolph et al., 2021). If we accept that differences exist between employees based on their birth year and/or period of socialization, then human resource (HR) management may need to differ in content and strategy to meet the needs of each generation, making general approaches to leadership impractical. For instance, if a particular generation does not prioritize relationships with colleagues, expects to achieve goals through close support from the organization, and/or tends to act independently, placing its members in work teams may not be the best decision. A study conducted by Bakanauskiene and Zagurskyte (2022), found that three generational cohorts differed in their happiness at work, in terms of how they prioritized expectations, types of relationships, and personal values. Decisions based on generational differences are welcome in HR management because they match the way people naturally categorize others. According to social identity theory (Tajfel and Turner, 1979), individuals quickly categorize themselves and others into different social groups (e.g., by age, nationality, profession), which makes the social world easier to understand. Therefore, categorization enables managers to process complex information about their diverse workforce by identifying shared patterns. However, it is important to note that while classification is a natural cognitive tool, it must be used with caution, as the common stereotype that generational differences seem to predict societal diversity is not always empirically supported (Moore and Krause, 2021). The validity of generational theory is fundamentally compromised by the lack of consensus regarding the chronological boundaries that define each cohort. The definition of the time periods for each cohort is controversial and highly dependent on historical events, cultural context and the scope of the proposal. As Costanza and Finkelstein (2015) stated, “Stereotypes about generational differences in the workplace are unfounded and unwise” (p. 321). The authors argue that many generational differences are actually “age effects” or “period effects” rather than permanent generational traits. The true utility of these classifications lies in using them as a starting point to foster empathy toward a cohort that shares certain cultural experiences and facts, rather than as a rigid set of rules. In Gurbuz and Aytekin's study, for example, the generational-based stereotype was not supported in a sample of 427 U.S. employees. Furthermore, they found that despite globalization -that propels us to the so-called *melting pot* (Zangwill, 1909), the original classification of U.S. generations could not be generalized to the Turkish business context as expected (Gurbuz and Aytekin, 2020). The most compelling evidence was presented in a meta-analysis and qualitative review by Rudolph et al. (2021), which systematically deconstructs the concept of generations in the workplace. The review concludes that generational differences are largely a myth with little to no empirical support. By synthesizing data from over 100 independent studies (covering nearly 100,000 participants), the researchers showed that differences in job satisfaction, engagement, and turnover intentions were driven by age and life stage rather than birth cohort. They revealed that generational membership explained less than 2% of the variance in worker attitudes. Furthermore, the authors warn that relying on generational labels can promote harmful stereotypes and ageism, recommending that organizations shift toward a lifespan perspective that treats employees as individuals based on their specific career stages and needs (Rudolph et al., 2021).

Entering the polarized debate surrounding age-based demographics, this study examines the empirical validity of generational classifications within the Spanish workforce. We challenge the effectiveness of ‘one-size-fits-all' HR strategies by comparing the predictive power of generational cohorts *vs*. individual variables. This paper aims to achieve a distinct original contribution to the field of organizational psychology by providing empirical evidence that calls into question the practical relevance of generational determinism. Furthermore, our data suggest that organizational HR strategies should focus on life stages and individual circumstances rather than broad generational labels, and may therefore contribute to the scientific debate.

### 1.1 Generational cohort definition and classifications

A generation is typically defined as a group of people born at a specific time who share similar experiences, significant life events, and historical contexts during the critical developmental years of their lives, and whose values and priorities tend to remain relatively stable throughout their lives (Clements, 2025). Generational theory defines it as a group of individuals with an average age of 25–30 years, referred to in the social science as a ‘line' (i.e., a sequence of species, each of which is thought to have evolved from its predecessor). Generations are created and shaped by external influences such as the media, economic and social events, popular culture, and the values shared by family and friends that guide their behavior (Parry and Urwin, 2021). Therefore, it is essential to acknowledge the concept of generation and to revise accepted classifications of generational cohorts. Sociological research has identified specific patterns of values and attitudes that is associated with a particular age groups within a homogeneous age spans that can be defined in isolation. According to Rudolph et al. (2021) generations are shaped by a particular period of time, which gives their members a collective persona. While there was consensus on including the most recent generations since the end of the First World War in five groups, defining the period for each group was more controversial and depended on historical events, context, culture, region, and the scope of the proposal. As illustrated in Table 1, experts who categorize typologies in terms of generations typically assign the Baby Boomer generation to the 1940s, Generation X to the 1960s, Generation Y (also known as Millennials), to the 1980s, Generation Z (also known as Centennials), to the 1990s, and the most recent Generation alpha to the 2000s.

**Table 1:** Recent proposals for a generation typology from 1940.

**Years**	**Tapscott, 1998**	**McIntosh-Elkins et al., 2007**	**Gibson et al., 2009**	**Dejnaka, 2015**	**Fry and Parker, 2018**	**Betz, 2019**
1940…						BB
…1946	BB	BB	BB	BB	BB	BB
…1962	BB	BB	BB	BB	BB	BB
1963	BB	X	BB	X	BB	BB
1964	BB	X	BB	X	BB	BB
1965	X	X	X	X	X	X
…1977	Y	X	X	X	X	X
1978	Y	Z	X	Y	X	X
1979	Y	Z	X	Y	X	X
1980	Y	Z	X	Y	Y	Y
…1986	Y	Z	Y	Y	Y	Y
…1994	Y		Y	Y	Y	Y
1995	Y		Y	Y	Y	Y
1996	Y		Y	Y	Y	Z
1997	Y		Y	Y	Y	Z
1998			Y	Z	Y	Z
…2000			Y	Z	Y	Z
…2007				Z		Z
…2009				Z		Alpha
2010				Z		Alpha
2012…						Alpha

Source: Own elaboration. BB, Baby Boomer; X, Generation X; Y, Millennials; Z, Generation Z; Alpha, Generation Alpha.

This study reviews the generational typology published by the Pew Research Center in 2019. For decades, this Washington-based organization has been a leader in measuring generational shifts through public opinion polling, demographic research, and content analysis. With over 39,400 citations on Google Scholar since 1940, the Center's data-driven approach highlights that generational cohorts are shaped by significant historical shifts rather than uniform chronological spans. For instance, baby boomers—the only generation officially designated by the US Census Bureau—are those born between 1946 and 1964; Generation X falls between 1965 and 1980; Generation Y follows, from 1981 to 1996; and Generation Z includes those born between 1997 and 2012. Under this framework, Generation Alpha remains excluded from current work-engagement analyses due to their age. Nevertheless, cultural constraints may influence the choice of generational typology. Rather than allowing for fixed time cycles between generational emergence, historical landmarks may serve as generational boundaries, and societal changes across generations should be considered in relation to these. Some of these landmarks may have varying impacts on society across different time spans. Accordingly, generational typologies depend on a country's history or culture and cannot be generalized (Cho et al., 2025; Gurbuz and Aytekin, 2020). Although globalization reduces cultural cues worldwide (Kasiyarno and Apriyanto, 2025), events that influence generational development in American society may not be applicable to other countries, such as Spain. From a historical materialist perspective (Ashley, 1982), the social and technological impacts on societies could be an appropriate way to analyse generational evolution in terms of lineage, i.e., significant social and cultural evolution between generations.

The Pew Research Center (2019) typology is theoretically and methodologically justified in this study by its emphasis on “historical anchors” rather than arbitrary fixed-year intervals. Unlike purely mathematical classifications, this framework delineates cohorts based on shared formative experiences—such as significant technological shifts, socioeconomic trends, and geopolitical events—thereby providing a sociologically grounded lens for analysis. Furthermore, its pervasive usage in global academic literature establishes a standardized common language ensuring that the study's defining parameters remain comparable with those of broader organizational research and mitigating the ambiguity often found in ad-hoc generational definitions.

### 1.2 Historical Spanish landmarks within PEW classification

If we were to apply the generational years defined by the Pew Research Center to the Spanish context, a series of significant events could be identified for each Pew cohort.

- Baby Boomers (1946–1964): In Spain, this generation is not defined by the post-WWII victory experienced in the US, but by emerging from post-Civil War autarchy and the subsequent economic boom known as ‘Desarrollismo'. Those born during the years of international isolation and rationing experienced the harshness of the dictatorship first-hand, while those born later came of age during the pivotal Stabilization Plan of 1959, which opened up the Spanish market economy. Consequently, this generation became the primary workforce behind the ‘Spanish Miracle' of the 1960s, a period characterized by massive rural-to-urban migration and the rise of the middle class. They witnessed a dramatic transition from scarcity to the consumerism represented by the iconic SEAT 600, which may symbolize the onset of a mild ‘Spanish dream'.
- Generation X (born 1965–1980): In Spain, Generation X is known as the ‘Generation of the Transition', as they bridged the gap between the authoritarian regime of the past and modern democracy. Although they experienced the dictatorship during their early childhood, they came of age with the death of Francisco Franco in 1975 and the ratification of the 1978 Constitution, which established the legal framework for their adulthood. This generation fueled the cultural explosion known as ‘La Movida Madrileña' in the 1980s, embracing countercultural freedom and hedonism. They were also the first to experience significant social shifts, such as rapid secularization and the legalization of divorce in 1981, which coincided with a rise in both divorce rates and maternal employment among U.S.
- Generation Z (born 1997–2012): Spanish Generation Z is characterized as the ‘Crisis and Digital Generation', having grown up in an era of permanent connectivity and significant economic volatility, with no memory of the peseta currency. Their worldview was initially shaped by the introduction of the euro in 2002, the geopolitical shock of the 11 March train bombings in 2004—not far from the 9/11 attacks in the US that transformed geopolitics and security worldwide—and most profoundly by the great recession (2008–2014) and the subsequent austerity measures that defined their childhoods. This economic backdrop, combined with the political awakening of the 15-M Movement (Indignados) in 2011, has produced a generation characterized by political skepticism, the fragmentation of the traditional two-party system and native fluency in digital environments (Digital Future Society, 2024).

While some historical landmarks in both countries bear similarities, the classification imposed from 1965 onwards imposes a ‘mathematical symmetry' (16-year blocks) upon sociological processes that are neither inherently linear nor symmetrical. This presents generations as merely methodological constructs or prefabricated boxes that attempt to impose order on a far more fluid reality. Ultimately, this approach loses predictive capacity when compared to continuous variables such as exact age. The artificiality of this symmetry in the definition of generational cohorts may hinder the usefulness of generational theory.

### 1.3 Generation cohorts, engagement, and the JD-R model

One of the key concepts that can differentiate companies and generate a competitive advantage is work engagement. Not only can this lead to higher employee performance, it can also create enthusiastic, engaged employees who are willing to share and generate new knowledge (Nguyen et al., 2024). Consequently, engagement is considered a predictor of significant outcomes for employees, work teams and organizations in the business sector (Adamu et al., 2025; Bakker and Albrecht, 2018). Engagement originates from positive psychology and has been defined as 'a positive, satisfying work-related state of mind characterized by vigor, dedication, and absorption' (Schaufeli et al., 2002). Rather than being a specific and momentary state, engagement refers to a more sustained and influential affective-cognitive state that is not focused on a particular object, event, person or behavior. Schaufeli et al. (2002) define these three components as follows:

- Vigor: an energetic and resilient state of mind, characterized by a willingness to invest time and effort in work, and to persevere despite difficulties.
- Dedication: experiencing a sense of excitement, inspiration, and challenge, while maintaining a positive attitude toward change. This goes beyond simple involvement, indicating a remarkably high level of participation.
- Absorption: characterized by deep concentration on work, where time passes quickly and it is difficult to disengage from the task. High levels of energy and a strong identification with work are therefore characteristic of engagement. All three components must be present simultaneously, although previous studies have shown that their degree of presence can vary.

For example, Castellano et al. (2019) and Lisbona et al. (2018) reported higher levels of vigor and dedication than absorption. However, other studies have found that only one dimension seems to dominate work engagement: dedication (Listau et al., 2017) or absorption (Shekari, 2015). Grant (2024) and Jones et al. (2018) argue that different generational cohorts have different perceptions of work and work engagement. Baby boomers prioritize professional dedication, value effective communication and seek personal fulfillment through their work. In contrast, Generation X views work as a contract prefers direct communication and is often skeptical of management (Grubb, 2023). Millennials are the most tech-savvy, educated and well-traveled generation in history (Hatice, 2024). They are high-maintenance employees who seek constant reassurance, feedback and support; however, they also value autonomy in the workplace (Chopra and Bhilare, 2020). The youngest generation, Centennials (also known as Generation Z), are technologically literate (Giray, 2022), entrepreneurial (Magano et al., 2020), independent (Jenkins, 2018), multitasking and pragmatic (Dorsey, 2016). They are also known for being highly customisable (Stillman and Stillman, 2017), and open-minded (Oxford Royale Academy, 2018). Studies on work engagement have found similarities and differences between members of different generations and within the same generation (Gurbuz and Aytekin, 2020; Suomäki et al., 2019).

For example, Havens et al. (2013) found no statistically significant differences in work engagement between cohorts, except for the absorption component, in which veterans scored higher than Generation X members.

Considering the lack of consensus in previous studies, this study establishes a critical theoretical dialogue between the Job Demands-Resources (JD-R) model and generational theory. Rather than complementing generational theory, the JD-R model is used as a framework to deconstruct and challenge its validity. Developed in the early 2000s by researchers Arnold Bakker and Evangelia Demerouti (along with colleagues such as Wilmar Schaufeli), the JD-R model is a comprehensive framework for explaining employee well-being (Bakker and Demerouti, 2007). It proposes that all working conditions can be categorized as either job demands (aspects requiring sustained effort that can lead to burnout) or job resources (aspects that help to achieve goals and stimulate personal growth). The model ultimately posits two simultaneous processes: a health impairment process driven by excessive demands and a motivational process driven by the availability of resources that fosters work engagement.

Therefore, differences in observed work engagement among employees may be better explained by the dynamic balance of job resources than by static generational traits. Rather than attributing these differences mainly to shared generational socialization, the model explains them in terms of structural conditions, resource accumulation and professional maturation. For instance, whereas traditional generational theory might attribute higher vigor scores to Baby Boomers due to a shared ‘work ethic', the JD-R model contextualizes this in terms of tenure-based resource accumulation. This suggests that engagement is driven by structural advantages associated with seniority, such as higher organizational status, job stability, and established professional networks, rather than unique generational personality traits. Consequently, the JD-R model may provide a simpler yet robust explanation for these differences based on unequal access to workplace resources.

The primary aim of this research was therefore to determine whether generational classifications serve as meaningful predictors of work engagement, and to test the discriminatory power of generational theories and classifications in contrast with the JD-R model.

The specific objectives of the research are:

**Objective 1:** To examine the relationship between generational cohorts (Baby Boomers, Generation X, Millennials, and Generation Z) and work engagement in Spain, with a specific analysis of the three dimensions of engagement: vigor, dedication and absorption.

**Objective 2:** To assess whether key sociodemographic variables, such as gender, occupation, and marital status, have a stronger association with work engagement and its dimensions than age.

**Objective 3:** To determine whether year of birth, when treated as a continuous variable, is a better predictor of work engagement than predefined generational categories.

This study examines the relationship between generational cohorts and work engagement in Spain, focusing on the three dimensions of engagement: vigor, dedication, and absorption. It aims to determine whether other sociodemographic factors are more strongly associated with engagement than age, and to assess whether a person's year of birth is a more effective predictor of engagement than predefined generational labels.

## 2 Method

### 2.1 Population

The study population consisted of adults who were born and were living in Spain. Initially, 475 people responded using non-probabilistic snowball sampling. Due to missing values for the year of birth, country of birth (other than Spain) and marital status, 68 participants were excluded. Work engagement, as operationalized with the UWES (The Utrecht Work Engagement Scale) (Schaufeli et al., 2002), is conceptualized as a work-related state, and its items are designed to be answered with reference to an ongoing job context. Including unemployed respondents would have necessitated different instructions (e.g. referring to a previous job) and could have led to increased measurement heterogeneity. Consequently, the final analyses focus on participants who are currently employed. Finally, two subjects were identified as multivariate outliers using Mahalanobis (1936) distance computed on the UWES dimension scores (vigor, dedication, and absorption). A conservative chi-square criterion was applied to determine the cut-off (*p* < 0.001; df = 3), and participants exceeding this threshold were excluded prior to the main analyses to reduce the potential influence of extreme multivariate response patterns. The final sample size was composed of 339 subjects, and the descriptive data are presented in Table 2.

**Table 2:** Descriptive data sample.

**Variable**	**Category**	**Boomers**	**X**	**Millennials**	**Z**	**Total**
Gender	Female	19	27	114	59	219
	Male	13	35	55	17	120
Marital status	Married	19	33	40	1	93
	Divorced	6	9	2	0	17
	Couple	3	5	57	14	79
	Single	4	15	70	61	150
Work status	Self-employed	3	8	2	0	13
	Employee	29	54	167	76	326

Source: own elaboration.

### 2.2 Measurement and instruments

The following socio-demographic data were collected from the participants: gender (male or female), year of birth, occupation (employed and self-employed); and generational affiliation, based on the Pew Research Center's (2019) classification: Baby Boomers (born 1946–1964), Generation X (born 1965–1980), Millennials (born 1981–1996), and Centennials (born 1997–2012). The Spanish version of the UWES-17 (the Utrecht Work Engagement Scale) (Schaufeli et al., 2002) was used to measure work engagement. This scale is the most widely used measure in this field, and has featured in 86% of studies on work engagement (Bailey et al., 2017; Byrne et al., 2016). It consists of 17 items with responses collected on a seven-point Likert scale, ranging from 0 (never) to 6 (always or every day). The UWES-17 scale calculates a total engagement score based on three factors: Vigor, Dedication and Absorption. The vigor factor (six items: 1, 4, 8, 12, 15, and 17) refers to having high levels of energy, resilience, willingness to put in effort, and perseverance in the face of difficulties. The dedication factor (five items: 2, 5, 7, 10, and 13) focuses on a high level of involvement in work, characterized by enthusiasm, inspiration, pride, and a sense of challenge. The Absorption component (six items: 3, 6, 9, 11, 14, and 16) measures complete immersion, attention, and concentration at work, where time passes unnoticed. Scale scores for the three subscales (Vigor, Dedication, and Absorption) and the total UWES (Utrecht Work Engagement Scale) score were calculated in accordance with the authors' instructions. The internal consistency of the UWES-17 and its three subscales has been widely examined and has been shown to be reliable. Cronbach's alpha values are generally reported to be in the range of 0.80–0.90 (Salanova et al., 2000).

### 2.3 Procedure

This quantitative, descriptive, cross-sectional study examined the relationship between generational cohorts, sociodemographic variables and work engagement. Work engagement was assessed using the UWES-17 questionnaire. The Job Demands–Resources (JD-R) model was employed as a broad theoretical framework to contextualize engagement as a motivational outcome. However, specific job demands and job resources were not directly operationalized or tested in the present study. Consequently, the analyses focused on age-based indicators (generational cohort and year of birth) and selected sociodemographic correlates (e.g., gender, occupation, and marital status) in relation to engagement. Data were collected via a Google Forms questionnaire between November 2022 and March 2023, using a non-probability snowball sampling method. While this approach facilitated rapid access to working adults across a wide age range, it also has well-known limitations. Firstly, as inclusion depended on personal networks and self-selection, the resulting sample may overrepresent individuals who are more digitally connected, more interested in the topic, or more willing to complete online surveys, while underrepresenting harder-to-reach workers. Secondly, snowball recruitment can amplify network homophily, whereby respondents tend to recruit others with similar sociodemographic or occupational characteristics, which could reduce heterogeneity. Consequently, the estimates reported here should be interpreted as associations within the observed sample rather than as population parameters. The external validity of generational comparisons may thus be limited, particularly given the uneven distribution of cohorts (e.g., the smaller Baby Boomer subsample). Therefore, while the findings presented here complement research based on probabilistic or stratified sampling of the Spanish workforce, they do not replace it.

In accordance with Spanish Organic Law 3/2018, of 5 December on the Protection of Personal Data and the Guarantee of Digital Rights, informed consent was provide from all participants. The link to the questionnaire was disseminated via email, social media, and smartphone applications (e.g., WhatsApp). In line with ethical standards, the research protocol took an average of seven minutes to complete. The collected data ensured participant anonymity and complied with ethical guidelines. No compensation was offered for participation.

## 3 Results

The collected data were analyzed using IBM SPSS Statistics Version 26 (IBM Corp, 2019). First, the reliability of the UWES-17 scale was determined using Cronbach's alpha analysis. The resulting score of 0.944 indicated excellent reliability, confirming the internal consistency of the UWES-17 scale and aligning with the findings of previous research. Next, the assumption of normality was assessed using the Kolmogorov-Smirnov test. This is an appropriate statistical method for datasets of more than 50 observations. The analysis revealed a non-normal distribution (*p* < 0.001), meaning that non-parametric tests had to be used for subsequent analyses.

### 3.1 Engagement and generation categories

The Kruskal–Wallis test revealed significant differences were observed between generations for the vigor factor (H = 9.516; *p* = 0.02). However, no such differences were found between generations for the dedication or absorption factors, or for the total engagement scale score (*p* > 0.05) (see Table 3). Previous studies have indicated that the emotional and motivational aspects of engagement are more susceptible to generational values and socialization processes, whereas the behavioral and cognitive dimensions are influenced more by situational and structural factors that are common to all age groups (Bakker and Demerouti, 2007; Schaufeli et al., 2002; Twenge, 2017). Vigor is more closely associated with affective and motivational orientations, which are significantly influenced by generational socialization, career expectations and values. The emphasis placed on enthusiasm, emotional expression, and intrinsic motivation in work or activities differs between generations, resulting in observable variations in vigor (Schaufeli et al., 2002; Singh et al., 2021). Conversely, dedication and absorption are behavioral and task-oriented dimensions of engagement that may be less sensitive to generational value differences and more strongly influenced by situational or organizational factors shared across age groups (Decuypere and Schaufeli, 2019; Schaufeli et al., 2006). Consequently, intergenerational similarities in roles, responsibilities and structural conditions may contribute to comparable levels of dedication and absorption, despite evident differences in affective engagement. This pattern suggests that generational differences may be more evident in how individuals feel about engagement than in how consistently or intensely they act on it.

**Table 3:** Engagement and factors by generation categories.

	**Variable**	***M***	***SD***	***H***	***Sig*.**
	Engagement	4.117	1.133	5.141	0.162
All generations	Vigor	4.210	1.119	9.516	0.023^*^
	Absorption	3.857	1.162	3.544	0.315
	Dedication	4.285	1.330	5.198	0.158

^*^p < 0.05.

M, Mean; SD, Standard Deviation; H, Kruskal-Wallis test value.

To understand potential variations in the vigor factor across generations, the Mann-Whitney U test was employed to compare each pair of intergenerational groups, as detailed in Table 4.

**Table 4:** Vigor factor by generation.

**Comparison**	**Group 1 *M (SD)***	**Group 2 *M (SD)***	***U***	***Sig*.**
Baby Boormers vs. Generation X	3.84 (1.09)	4,44 (1.13)	647.5	0.006^**^
Baby Boormers vs. Millennials	3.84 (1.09)	4.17 (1.12)	2,128.5	0.056
Baby Boormers vs. Generation Z	3.84 (1.09)	4.25 (1.06)	925.5	0.05^*^
Millennials vs. Generation X	4.17 (1.12)	4.44 (1.13)	4,309	0.038^*^
Millennials vs. Generation Z	4.17 (1.12)	4.25 (1.06)	6,141.5	0.58
Generation X vs. Generation Z	4.44 (1.13)	4.25 (1.06)	2,012.5	0.14

^*^p ≤ 0.05.

^**^p < 0.01.

M, Mean; SD, Standard Deviation; U, Mann-Whitney U-test value.

The detailed results reveal some notable patterns. A significant difference was found in the vigor factor between baby boomers and generation X, with generation X showing a higher mean score (4.44) than baby boomers (3.84) (U = 647.5, *p* < 0.01). However, there was no statistically significant difference between Baby Boomers and Millennials (*p* > 0.05). When comparing Baby Boomers with Generation Z, Generation Z again performed better, with mean scores of 4.25 and 3.84 respectively (U = 925.5, *p* < 0.05). Focusing on Generation X vs. Millennials, Generation X performed better on the Vigor factor, with a mean score of 4.44 compared to 4.17 for Millennials (U = 4,309, *p* < 0.05). Subsequent analyses of the data comparing Generation X and Generation Z revealed no significant difference in vigor (*p* > 0.05). Similarly, no significant difference was found between Millennials and Generation Z on this factor (*p* > 0.05). These findings clearly illustrate how the vigor factor is experienced by different generational cohorts, contributing to a comprehensive understanding of work engagement dynamics across age groups. Following the assessment of intergroup differences, the effect size of the generational variable on the vigor factor was analyzed. Although the generational effect on vigor was statistically significant, the associated effect size was negligible. The squared Epsilon value (ER^2^ = 0.028) indicates that generational membership accounts for only 2.8% of the variation in vigor scores. In practical terms, while differences in vigor can be detected between generations at a statistical level, generation alone appears to be a relatively weak explanatory factor. This suggests that other variables, such as contextual, organizational or individual factors, are more likely to influence vigor than generational belonging itself.

### 3.2 Engagement and sociodemographic variables

#### 3.2.1 Gender

The Mann-Whitney U test was used to assess the relationship between gender and the variables of engagement, vigor, dedication, and absorption variables. The analysis revealed no significant genders differences between for any of these factors, as indicated by *p*-values greater than 0.05 (Table 5).

**Table 5:** Engagement and factors by gender.

**Variable**	**Gender**	***M***	***SD***	***U***	***Sig*.**
Engagement	Female	4.10	1.09	12,695.5	0.60
	Male	4.13	1.20		
Vigor	Female	4.19	1.08	12,507.5	0.46
	Male	4.23	1.18		
Absorption	Female	3.83	1.12	12,488.5	0.45
	Male	3.89	1.23		
Dedication	Female	4.28	1.31	13,137.5	0.99
	Male	4.27	1.37		

M, Mean; SD, Standard Deviation; U, Mann-Whitney U-test value.

#### 3.2.2 Occupation

Using the Mann-Whitney U-test to examine the relationship between occupation and commitment, vigor, dedication, and absorption, the following results emerged (Table 6).

**Table 6:** Engagement and factors by occupation.

**Variable**	**Occupation**	***M***	***SD***	***U***	***Sig*.**
Engagement	Employed	4.08	1,13	1,231	0.010^*^
	Self-employed	4.84	0.66		
Vigor	Employed	4.19	1.12	1,630.5	0.15
	Self-employed	4.69	0.7		
Absorption	Employed	3.81	1.16	814	0.000^**^
	Self-employed	4.88	0.44		
Dedication	Employed	4.25	1.33	1,406.5	0.039^*^
	Self-employed	4.96	1.02		

^*^p < 0.05.

^**^p < 0.01.

M, Mean; SD, Standard Deviation; U, Mann-Whitney U-test value.

A significant difference in engagement was found in favor of the self-employed. The effect size (Z^2^) for these differences was 0.019, indicating that occupation can explain 1.9% of the variability in engagement scores. No significant differences were found in the analysis for vigor (*p* > 0.05). In the case of dedication, however, a significant difference based on occupation emerged. The effect size (Z^2^) for these differences is 0.012, suggesting that occupation may account for 1.2% of the variability in dedication scores. Finally, a significant difference was observed in Absorption in relation to occupation. The effect size (Z^2^) for these differences is 0.042, suggesting that occupation can explain 4.2% of the variability in absorption scores.

#### 3.2.3 Marital status

The Kruskal-Wallis H-test revealed significant differences (*p* < 0.05) between marital status and overall engagement and its three factors: vigor, dedication, and absorption (see Table 7).

**Table 7:** Engagement and factors by marital status.

**Variable**	**Marital status**	***M***	***SD***	***H***	***Sig*.**
Engagement	Singles	4.07	1.13	12.56	0.006^**^
	Coupled	4.02	1.11		
	Married	4.37	1.10		
	Divorced	3.53	1.09		
Vigor	Singles	4.14	1.10	13.77	0.003^**^
	Coupled	4.11	1.11		
	Married	4.48	1.13		
	Divorced	3.75	0.93		
Absorption	Singles	3.81	1.18	9.59	0.02^*^
	Coupled	3.76	1.11		
	Married	4.11	1.12		
	Divorced	3.28	1.22		
Dedication	Singles	4.25	1.34	9.83	0.02^*^
	Coupled	4.20	1.32		
	Married	4.53	1.26		
	Divorced	3.55	1.35		

^*^p < 0.05.

^**^p < 0.0.

M, Mean; SD, Standard Deviation; H, Kruskal-Wallis test value.

Specifically, married individuals consistently showed higher engagement than other groups. Compared to single individuals, married participants had significantly higher engagement scores (mean = 4.37 vs. 4.07; U = 5,818.5, *p* < 0.05), vigor scores (mean = 4.48 vs. 4.14; U = 5,461, *p* < 0.01) and absorption scores (mean = 4.11 vs. 3.81; U = 5,913.5, *p* < 0.05). However, no difference was found for dedication. A similar pattern emerged when married individuals were compared to those in couples, with the former scoring higher on engagement (mean = 4.37 vs. 4.02; U = 2,904, *p* < 0.05), vigor (mean = 4.48 vs. 4.11; U = 2,878.5, *p* < 0.05) and absorption (mean = 4.11 vs. 3.76; U = 2,961.5, *p* < 0.05). The most significant differences were observed when comparing married and divorced individuals. Married participants achieved higher scores on all four factors: engagement (mean = 4.37 vs. 3.53; U = 409.5, *p* < 0.01), vigor (mean = 4.48 vs. 3.75; U = 439, *p* < 0.01), dedication (mean = 4.53 vs. 3.55; U = 437, *p* < 0.01) and absorption (mean = 4.11 vs. 3.28; U = 480, *p* < 0.01). Conversely, single individuals scored higher than their divorced counterparts on engagement (mean = 4.07 vs. 3.53; U = 891, *p* < 0.05) and dedication (mean = 4.25 vs. 3.55; U = 863, *p* < 0.05). The effect size (ER^2^) of marital status on the variables ranged from 0.028 to 0.041, indicating that marital status accounts for approximately 3% of the variability in the test scores.

These findings suggest that marital status may influence how engaged individuals are, with married people benefiting from greater social and emotional stability. Conversely, divorced individuals may experience resource depletion, a pattern consistent with the Job Demands–Resources model and work–family resource perspectives (Bakker and Demerouti, 2007; ten Brummelhuis and Bakker, 2012; Zacher and Rudolph, 2022).

### 3.3 Discriminant analysis of generational categories

Following the findings, a discriminant analysis was conducted to explore the influence of the controlled variables. This analysis used a four-rank generational category as the grouping variable and participants' year of birth and total engagement scores as the discriminant variables. The canonical discriminant function revealed canonical correlations of 0.102 and 0.944 for the engagement and year of birth variables, respectively. These results suggest that year of birth is the main factor in distinguishing between generational groups, whereas total engagement does not significantly predict these intergenerational distinctions.

## 4 Discussion

This study examined the complex relationship between different generational cohorts and the work engagement of adults born and living in Spain, focusing on vigor, dedication, and absorption. The results highlighted notable generational variations, particularly within the vigor factor, in line with existing literature on the multifaceted nature of work engagement in organizational settings. Unlike some studies which reported no age-related differences in engagement (Salas-Vallina and Alegre, 2017), these findings identified nuanced associations between age and particular engagement facets, consistent with the work of Bezuidenhout and Cilliers (2011). Recent research suggests that generational differences are more likely to manifest in affective and motivational orientations (e.g., passion) than in task-focused engagement behaviors such as dedication and absorption (Rudolph et al., 2021; Schaufeli, 2021). Our results align closely with those of the comprehensive meta-analysis conducted by Rudolph et al. (2021). The 2.8% variance in vigor that is explained by generational membership is consistent with their finding that cohorts account for less than 2% of worker attitudes. This supports the idea that generational labels are less empirically substantial than life-stage factors. Although the generational effect on vigor was statistically significant, it explained only a small proportion of variance. This pattern aligns with an expanding body of organizational and HR research that challenges the substantive value of generational labels and warns against generational essentialism—the belief that people born in specific years inherently possess distinct psychological or behavioral traits (Parry and Urwin, 2021; Rauvola et al., 2019). Consequently, the present findings contribute to this broader critique by demonstrating that generational differences, even when statistically detectable, account for minimal variance in engagement outcomes. This highlights the need for more nuanced, context-sensitive approaches in organizational research and practice, rather than focusing on generational typologies. Examining the vigor factor revealed interesting patterns: Generation X and Generation Z scored significantly higher than Baby Boomers, suggesting a trend toward greater energy and resilience among younger workers. However, no significant differences were observed between Millennials and Baby Boomers, highlighting the diversity within each generation. Beyound generational influences, certain sociodemographic variables were found to be significantly associated with work engagement. Occupation was identified as a key determinant, with self-employed individuals reporting higher levels of engagement. Marital status was also found to be significantly associated with work engagement, with married respondents consistently exhibiting greater engagement than their single or divorced counterparts. These factors impact motivation and the balance between professional and personal life. From the perspective of Self-Determination Theory (SDT) (Ryan and Deci, 2000), engagement is fostered when employees experience satisfaction of basic psychological needs—autonomy, competence, and relatedness. These needs can be supported by stable social relationships and meaningful roles outside work. Research applying SDT to workplace outcomes has found that autonomous motivation and need satisfaction are positively associated with engagement and well-being (McAnally and Hagger, 2024), suggesting that individuals in supportive personal circumstances, such as those who are married, may experience greater psychological resources that carry over into work. Conversely, individuals undergoing life transitions, such as divorce, may experience need frustration or reduced social support, which can potentially diminish their capacity for engagement. Marital status is also relevant in the work–life balance literature, which highlights the interplay between work and family roles and how effective balance can enhance engagement. Employees who successfully integrate work and family demands often report higher engagement, as work–life balance has been shown to positively relate to psychological capital and overall engagement to work (Wood et al., 2020). Similarly, self-employed individuals often enjoy greater autonomy and control over their work, which are key facilitators of intrinsic motivation and engagement according to SDT and job design research (Wuisan et al., 2025). Consequently, marital and employment status can be considered as contextual resources that influence engagement by shaping motivational processes and the ability to balance personal and professional roles. A discriminant analysis further highlighted the dominant influence of year of birth over engagement level in distinguishing between generational cohorts, a finding that adds depth to existing literature. These findings suggest that generational cohort membership primarily reflects socio-historical positioning rather than meaningful differences in engagement levels. In line with prior research, the results reinforce critiques of generational essentialism, suggesting that birth year is a categorization marker rather than a significant predictor of workplace attitudes or behaviors (Costanza and Finkelstein, 2015; Parry and Urwin, 2021; Rudolph et al., 2021). The minimal explanatory power of engagement underscores the greater relevance of contextual and situational factors over generational labels. From a theoretical perspective, engagement-related motivational processes, as described in SDT, appear to operate similarly across cohorts. This reinforces the view that age, career stage, and socio-economic context are more influential determinants of engagement than generational membership alone (McAnally and Hagger, 2024).

Overall, the present study makes three significant contributions to research on work engagement research. Firstly, it shows that an individual's year of birth is a more reliable predictor of work engagement than broad generational categorizations. This raises question about the continued use of static generational cohorts in HR management. Secondly, the research highlights the greater influence of sociodemographic factors, such as marital status and occupation, as married individuals and the self-employed report higher levels of engagement. Thirdly, the findings refine existing generational frameworks by showing that generational membership accounts for minimal variability in engagement scores. This emphasizes the need for a more personalized approach to understanding and improving employee engagement.

## 5 Conclusions

A key conclusion is that the significant differences in vigor scores between generations suggest that work-related energy and resilience are experienced differently by each cohort. The higher vigor scores observed in Generation X and Z compared to the Baby Boomers suggest a potential shift in work attitudes over time. Furthermore, the study also confirms the significant impact of sociodemographic variables, specifically occupation and marital status, on engagement levels. The higher engagement levels observed among the self-employed individuals highlight the importance of autonomy and control. The consistent findings for married individuals also emphasize the need to consider personal circumstances in corporate initiatives. Ultimately, the discriminant analysis reinforces the idea that historical and contextual factors tied to a person's birth year play a greater role in shaping broad work attitudes than engagement levels themselves. This suggests that a “one-size-fits-all” approach to engagement may be ineffective. To foster a truly inclusive and high-performing workplace, organizations need to adapt their strategies to reflect the diverse needs and values of their workforce.

From an applied perspective, HR professionals may use marital and employment status as contextual indicators (rather than determinants) when tailoring engagement-supportive practices. For example, providing stronger work–life and social support resources (e.g., flexible working hours, consistent scheduling and mentoring) and ensuring equitable access to onboarding, supervisor support, training and career opportunities—especially for those in more precarious situations—may help to maintain engagement.

The study's cross-sectional design is its primary limitation, since it cannot establish cause-and-effect relationships. Future research employing a longitudinal design would be better able to track the evolution of work engagement throughout an individual's career. Additionally, the use of self-reported data introduces the potential for common method bias and social desirability effects, whereby participants may answer in a way that they believe to be socially acceptable. Future studies could use a multi-method approach, such as combining surveys with observational data or objective performance metrics, to mitigate this issue. Researchers should also consider controlling for social desirability to ensure more accurate and reliable results.

The findings are also limited by the use of a non-probabilistic snowball sampling strategy. In addition to limiting generalizability, snowball sampling may introduce selection and coverage biases due to reliance on participants' social networks and online dissemination channels. Consequently, some segments of the Spanish workforce may be systematically underrepresented (e.g., those with limited digital access or those outside the reached networks), while clusters of demographically similar respondents may be overrepresented. This is particularly relevant when examining age-related patterns, as cohort sizes are imbalanced. Future studies should adopt probabilistic or quota-based sampling approaches (e.g., stratification by age group, sector, and region) to enhance representativeness and assess whether the observed patterns hold under conditions of stronger external validity.

Additionally, the analytical sample was restricted to participants who were currently employed, as unemployed respondents were excluded during the data cleaning to ensure consistency with the construct being measured (i.e., work engagement, as assessed using the UWES, which assumes the respondent has a current work role). While this decision strengthens the interpretability of the engagement scores within an active employment context, it also limit the scope for inference. Thus, the conclusions should be interpreted as applying to the employed Spanish workforce, rather than to unemployed individuals or to adults outside the labor market, whose work-related psychological experiences may differ substantially during periods of labor-market transition. Future research should explicitly incorporate employment status (including unemployment and job transitions) to examine whether the observed patterns are replicated across different labor market positions.

To develop a comprehensive understanding of work engagement, it is necessary to explore factors that were not included in this study, such as organizational culture, leadership style and job design. These variables are known to have a significant influence on employee engagement and could provide a richer context for the current findings. Finally, to address the assumption of homogeneity within generational cohorts, future research could examine variations within generations in relation to specific cultural, occupational or organizational factors. This would provide a more nuanced understanding of how engagement can differ even among individuals within the same generation.

This research confirms that generational labels should be treated as descriptive cohort markers rather than deterministic predictors of workplace behavior, since they conflate the effects of age, period and cohort. Future research should complement generational categorizations by using age as a continuous indicator, as well as considering more proximal explanatory variables such as life stage, tenure, employment conditions and job demands/resources. Where possible, longitudinal or cohort-sequential designs should be used, along with robustness checks, to clarify whether any observed differences persist beyond categorization choices.

## Data Availability

The original contributions presented in the study are publicly available. This data can be found here: https://doi.org/10.6084/m9.figshare.25600617.
